# Glimepiride Promotes Osteogenic Differentiation in Rat Osteoblasts via the PI3K/Akt/eNOS Pathway in a High Glucose Microenvironment

**DOI:** 10.1371/journal.pone.0112243

**Published:** 2014-11-12

**Authors:** Pan Ma, Bin Gu, Wei Xiong, Baosheng Tan, Wei Geng, Jun Li, Hongchen Liu

**Affiliations:** 1 Department of oral implantology, Beijing Stomatological Hospital of Capital Medical University, Beijing, China; 2 Department of Stomatology, Chinese PLA General Hospital and Postgraduate Military Medical School, Beijing, China; 3 Clinical Aviation Medicine Center of PLA, Air Force General Hospital, Beijing, China; University of Udine, Italy

## Abstract

Our previous studies demonstrated that glimepiride enhanced the proliferation and differentiation of osteoblasts and led to activation of the PI3K/Akt pathway. Recent genetic evidence shows that endothelial nitric oxide synthase (eNOS) plays an important role in bone homeostasis. In this study, we further elucidated the roles of eNOS, PI3K and Akt in bone formation by osteoblasts induced by glimepiride in a high glucose microenvironment. We demonstrated that high glucose (16.5 mM) inhibits the osteogenic differentiation potential and proliferation of rat osteoblasts. Glimepiride activated eNOS expression in rat osteoblasts cultured with two different concentrations of glucose. High glucose-induced osteogenic differentiation was significantly enhanced by glimepiride. Down-regulation of PI3K P85 levels by treatment with LY294002 (a PI3K inhibitor) led to suppression of P-eNOS and P-AKT expression levels, which in turn resulted in inhibition of RUNX2, OCN and ALP mRNA expression in osteoblasts induced by glimepiride at both glucose concentrations. ALP activity was partially inhibited by 10 µM LY294002. Taken together, our results demonstrate that glimepiride-induced osteogenic differentiation of osteoblasts occurs via eNOS activation and is dependent on the PI3K/Akt signaling pathway in a high glucose microenvironment.

## Introduction

Type 2 diabetes mellitus (DM) is a metabolic disease with elevated morbidity and mortality. It is characterized by hyperglycemia secondary to peripheral insulin resistance, with a variable degree of hyperinsulinemia and insulin secretion impairment. Hyperglycemia has various adverse effects on bone metabolism, especially in patients with poorly controlled DM. Currently available data on bone metabolism and fracture risk in patients with DM are partly conflicting and inconclusive due to inhomogeneous study populations and designs. Patients with DM have various skeletal disorders, including osteopenia or osteoporosis, Charcot's arthropathy and the diabetic foot syndrome [1]–[3].

Glimepiride, a third generation sulfonylurea, exerts its effects primarily by stimulating insulin secretion, but has also been shown to have pleiotropic effects. In addition to its stimulatory effects on pancreatic insulin secretion, glimepiride has been reported to have extrapancreatic functions including activation of PI3K (phosphoinositide 3-kinase) and Akt(v-akt murine thymoma viral oncogene homologue) in rat adipocytes and skeletal muscle [4]–[6]. In endothelial cells, it is suggested that glimepiride induces endothelial nitric oxide synthase (eNOS) phosphorylation with a dependent mechanism of PI3K/Akt [7], [8]. Osteoblasts are bone forming cells that play an essential role in osteogenesis. Osteoblasts differentiate from mesenchymal stem cells and form bone through endochondral and intramembranous ossification. Many signaling molecules have been identified that positively or negatively regulate osteoblast differentiation. For example, PI3K/Akt signaling is crucial for osteoblast differentiation [9],[10], whereas p53 is a negative regulator of osteogenesis [11].

The PI3K/Akt pathway is involved in signal transduction related to cell growth, proliferation, differentiation, motility, survival and metabolism [12]. The protein kinase Akt, a multifunctional regulator of cell survival, is a downstream effector of PI3K. Recent studies suggest that Akt is activated by phosphorylation via activated PI3K and phosphorylates eNOS on serine 1177, thereby activating this enzyme. By inducing eNOS activity, the PI3K/Akt/eNOS pathway provides an enhanced cell survival signal [13], [14]. We previously demonstrated that glimepiride activates the PI3K/Akt pathway, and this activation is likely to be required for glimepiride to stimulate the proliferation and differentiation of rat osteoblasts [15], [16]. However, it remains unclear whether the PI3K/Akt/eNOS pathway can regulate osteoblastic cell differentiation following treatment with glimepiride in a high glucose microenvironment.

In the present study, we investigated whether glimepiride enhanced bone formation by osteoblasts in vitro and whether the effect was induced by up-regulation of eNOS through the PI3K/Akt pathway. Expression of P-eNOS (Ser1177) and P-Akt (Ser473) in a control group and a high glucose group were measured after stimulation with glimepiride. We found that glimepiride significantly enhanced the proliferation and differentiation of osteoblasts. Moreover, pre-administration of LY294002 (a highly specific, reversible inhibitor of PI3K) not only significantly reversed the differentiation ability of osteoblasts, but also inhibited the up-regulation of P-eNOS and P-Akt. Our findings suggest that glimepiride activates eNOS expression in rat osteoblasts via the PI3K/Akt pathway, and this activation is likely required for glimepiride to stimulate differentiation of rat osteoblasts in a high glucose microenvironment.

## Materials and Methods

### Study subjects

Sprague Dawley rats aged 6–8 weeks were purchased from the Laboratory Animal Center of the Academy of Military Medical Sciences (Beijing, China). We confirm that Military Medical Sciences Intramural Animal Use and Care Committee approved this study. All experiments were performed following the Guidelines of the Academy of Military Medical Sciences Intramural Animal Use and Care Committee.

### Cell culture

Primary osteoblasts were prepared and purified as described in our previous report [15], [16]. In brief, we minced the mandibles into fragments after removal of the condylar cartilage, connective tissue and alveolar sockets including the periodontal ligaments. The tissues were washed in sterile phosphate buffered solution (PBS) and then digested in a mixture of 3 mg/mL 0.1% collagenase type I (Gibco BRL, Gaithersburg, MD) for 30–40 min at 37°C. Single-cell suspensions were generated by filtration through a 100 mm strainer, washed, and resuspended in Dulbecco's modified Eagle's medium (DMEM; Gibco BRL) supplemented with 10% fetal bovine serum, 0.292 mg/mL of glutamine (Invitrogen, Carlsbad, CA), 100 U/mL of penicillin and 100 mg/mL of streptomycin (Gibco BRL) at 37°C in a humidified atmosphere of 5% CO_2_ and 95% air. Glimepiride was purchased from Sigma (St. Louis, MO). Osteoblasts were treated with 10 µmol/L glimepiride and/or 10 µmol/L LY294002 (Cell Signaling Technology, Danvers, MA) in 5.5 mM or 16.5 mM glucose [17]. The concentrations for glimepiride and LY294002 were chosen according to previous reports [4]–[6], [18]–[20]. The cells then were harvested and subjected to assays for the in vitro biological characteristics of osteoblasts. The same passage of cells was used for each experiment. For osteoblast differentiation, cells (2–5×10^3^/cm^2^) were incubated in DMEM containing 10% fetal calf serum (FCS) with 5.5 mM or 16.5 mM glucose. Cultures were fed every third day and characterized after 14 days by Alizarin Red S staining. To measure the amout of stain, the stain was solubilized with 0.5 mL of 5% sodium dodecyl sulfate (SDS) in 0.5 N HCl for 30 min at room temperature. Solubilized stain (0.15 mL) was transferred to a 96-well plate and absorbance was measured at 405 nm.

### Immunocytochemical and Immunofluorescence staining

Osteoblasts were seeded on coverslips in a 24-well plate and further cultured for 24 h before staining. After washing in PBS and fixing in 4% paraformaldehyde for 15 min, the samples were incubated with alkaline phosphatase (ALP), collagenase 1 (COL-1) and osteocalcin (OCN) (1∶200; R&D Systems Inc., Minneapolis, MN) for 2 h and subsequently incubated with fluorescein isothiocyanate (FITC)- or Rhodamine-conjugated antirabbit secondary antibodies. eNOS and P-eNOS (1∶100; Santa Cruz Biotechnology, Santa Cruz, CA) immunocytochemical staining was performed on osteoblasts that had been cultured in DMEM containing 10% FCS with 5.5 mM or 16.5 mM glucose. The osteoblasts were cultured in medium containing 5.5 mM or 16.5 mM glucose for 24 h and then changed to medium containing 10 µM LY294002 for 120 min. After being fixed in 4% paraformaldehyde, samples were incubated with e-NOS (1∶100; Santa Cruz) for 2 h and subsequently incubated with FITC-conjugated antirabbit secondary antibodies. Positive cells were observed under a fluorescence microscope (Olympus Optical, Tokyo, Japan). Each experiment was repeated at least three times.

### MTT assay

Osteoblasts were cultured in 96-well plates (10^3^ cells/well). An MTT assay was conducted after 7 days using a cell proliferation kit (Sigma) according to the manufacturer's protocol. After 48 h, the culture medium was replaced with 100 µL of MTT (0.5 mg/mL). The black crystals that formed after 2–3 h were dissolved with acidified isopropanol and absorbance was measured at 570 nm with a microplate reader (Bio-TEK Instruments, Winooski, VT).

### Determination of apoptosis

Apoptosis was measured by Annexin-V staining (Partec, Münster, Germany) according to manufacturer's instructions. After incubation with or without 5 µM 7-xylosyl-10-deacetylpaclitaxel for 48 h, cells were spun at 1200×g for 5 min and the supernatant was decanted. The cell pellet was resuspended with 100 µL of Annexin-V binding buffer and 5 µl of Annexin-V dye and was left in the dark at room temperature for 15 min. Following incubation, an additional 400 µL of Annexin-V binding buffer was added to each sample. Ten thousand cells were acquired and the apoptotic cell was quantification by flow cytometry and FloMax software (Partec). This test was repeated 3 times.

### Quantitative real-time polymerase chain reaction (PCR)

Total cellular RNA was extracted from the different groups of osteoblasts using TRIzol Reagent (Invitrogen). Approximately 2–5 mg of total RNA was converted to cDNA using a Super Script First Strand Synthesis Kit (Invitrogen, Carlsbad, CA). Real-time PCR reactions were performed using a QuantiTect SYBR Green PCR Kit (Toyobo, Osaka, Japan) and an Applied Biosystems 7500 Real-Time PCR Detection System. Two independent experiments were performed for each reaction in triplicate. The primers used are listed in Table 1.

**Table 1:** Primer sequences used in the reverse transcription polymerase chain reaction (RT-PCR).

Gene	Forward primer (5′-3′)	Reverse Primer(5′-3′)
CCND1	AAAGGCCAGTATGCACAGCTTTC	TTCAACCACTGGGCCACTATTTC
RUNX2	GATAACCTGGATGCCGTCGTG	CAGCCTAGCCAGTCGGATTTG
OCN	CAGCGTTATGAGATCAAGATGACCA	AGTGATGTGCAAGAGTCCATCCTG
ALP	GGAGCACTGTGTTTATGCTGGAA	GACCGAGCGATTGCTCAAGA
β-ACTIN	TGGCACCCAGCACAATGAA	GTCATAGTCCGCCTAGAAGCA

### ALP staining and ALP activity

Osteoblasts (10^4^/cm^2^) were seeded onto six-well plates, cultured in medium containing 5.5 mM or 16.5 mM glucose and stimulated with 10 µmol/L glimepiride and/or 10 µmol/L LY29400 induction medium. After 7 days, ALP activity was assayed using a BCIP/NBT alkaline phosphatase color development kit (Beyotime Institute of Biotechnology, Haimen, China) according to the manufacturer's protocol. For ALP staining, cells were fixed with 70% ethanol 7 days after induction and incubated with a solution of 0.25% naphthol AS-BI phosphate and 0.75% Fast Blue BB dissolved in 0.1 M Tris buffer (pH 9.3).

### Western blot analysis

Whole cell lysates were extracted with lysis buffer (10 mM Tris-HCL, 1 mM EDTA, 1% SDS, 1% Nonidet P-40, 1∶100 proteinase inhibitor cocktail, 50 mM β-glycerophosphate, 50 mM sodium fluoride) for western blotting. The protein content of the lysate was determined using a protein assay kit (Beyotime Institute of Biotechnology) following the manufacturer's recommended protocol. The proteins were loaded on 10% SDS polyacrylamide gels, transferred to PVDF membranes (Millipore, Bedford, MA) and blocked with 5% nonfat milk powder in PBS with 0.1% Tween. The membranes were probed overnight with the following monoclonal primary antibodies: anti-ALP, anti-Col-1, anti-OCN, anti-eNOS and anti-P-eNOS (Abcam, Cambridge, UK) and anti-Akt and anti-P-Akt and anti-PI3K P85 (Cell Signaling Technology, Beverly, MA). The membranes were then incubated with antimouse horseradish peroxidase-conjugated secondary antibody (Boster, Wuhan, China). The blots were visualized using an enhanced chemiluminescence kit (Amersham Biosciences, Piscataway, NJ) according to the manufacturer's recommended instructions. The ratio of the targeted band to actin was considered to be the relative intensity of the targeted band.

### Statistical analysis

For experiments with more than two groups, statistical analyses were performed using one-way ANOVA, and Bonferroni's method was applied to control for multiple testing. Statistical comparisons between two groups were performed by the Student's t-test. Differences were considered statistically significant at p<0.05.

## Results

### Glimepiride induced the proliferation and differentiation of rat osteoblasts in a high glucose microenvironment

**Figure 1:**
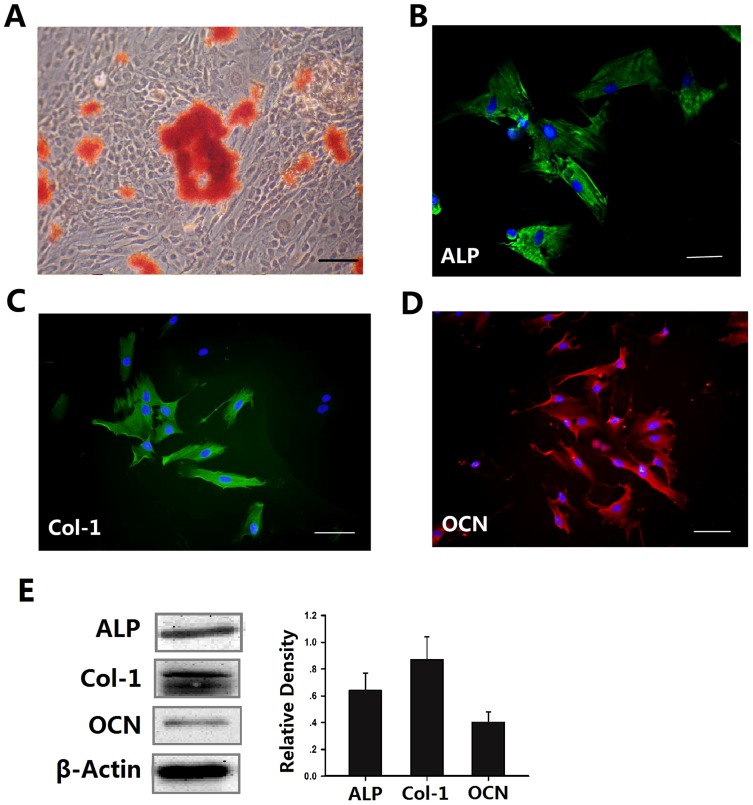
Verification of rat mandibular osteoblasts. **A**, Osteoblasts were cultured in DMEM containing 10% FCS for 14 days, then mineralized nodules were detected by Alizarin Red S staining. Immunocytochemical staining showed that the osteoblasts expressed ALP (**B**), Col-1 (**C**) and OCN (**D**). Scale bars represent 100 µm. The expression of ALP, Col-1 and OCN(E)were measured by Western blot in osteoblasts. β-Actin was used as control for equal loading. The graph shows the quantitative evaluation of the densitometric data of these proteins. The results represent mean ± standard deviation.

Osteogenesis is a complex process involving epithelial–mesenchymal interactions, condensation and differentiation. Metabolic disorders, including DM, are now well known to affect bone metabolism and may result in osteoporosis. To determine whether a high glucose microenvironment influences the biological characteristics of osteoblasts, and the effects of glimepiride (a third-generation sulfonylurea) on osteoblasts, we isolated osteoblasts from the mandible of rats. The isolated cells could differentiate and had formed distinct nodules after 14 days, as observed by Alizarin Red S staining. Dark red mineralized bone matrix (bone nodules) was visualized in stained sections (Fig. 1A). To identify osteoblasts, we detected their key proteins by immunofluorescence staining, which found the osteoblasts to be positive for ALP, COL-1 and OCN (Fig. 1B,C,D). Next we perform Western Blot to evaluated ALP,COL-1 and OCN expression in osteoblasts, we found that the osteoblasts were positive expression for ALP,COL-1 and OCN (Fig.1E).

**Figure 2:**
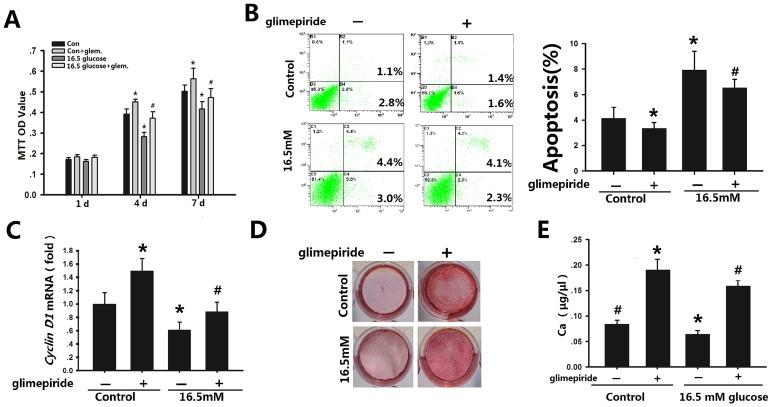
Effects of glimepiride on the proliferation and differentiation of rat osteoblasts. A, Effect of glimepiride on proliferation of rat mandibular osteoblasts incubated with two different glucose concentrations (MTT assay). Data represent mean ±SEM. ^*^p<0.05 vs. controls without glimepiride; # *p*<0.05 vs. 16.5 mM glucose without glimepiride. The statistical analysis were performed at day 1, 4 and 7. **B**, Apoptosis assay of control group (osteoblasts cultured with 5.5 mM glucose) and osteoblasts stimulated with 16.5 mM glucose with or without glimepiride treatment. The apoptotic cells were examined by using Annexin V staining in flow cytometric analysis. The apoptosis ratio was then calculated as shown in histogram. ^*^p<0.05 vs controls without glimepiride; # *p*<0.05 vs 16.5 mM glucose without glimepiride. **C**, Effects of glimepiride on cyclin D1 expression in osteoblasts cultured with 5.5 mM or 16.5 mM glucose for 120 min and then grown in basal medium for 48 h. Expression levels were normalized to that of GAPDH. ^*^p<0.05 vs controls without glimepiride; # *p*<0.05 vs 16.5 mM glucose without glimepiride. **D**, Photograph of culture wells showing Alizarin Red S-stained Ca^2+^ deposition from osteoblasts treated with glimepiride for 72 h and then cultured with 5.5 mM or 16.5 mM glucose for 14 days. **E**, Quantitative evaluation of Alizarin Red S staining. ^*^p<0.05 vs controls without glimepiride; # *p*<0.05 vs 16.5 mM glucose without glimepiride.

The control group proliferated faster than the group stimulated by 16.5 mM glucose (Fig. 2A). However, MTT assay showed that glimepiride promoted osteoblast proliferation not only in the control group but also in the 16.5 mM glucose group from day 4 to 7 (*p*<0.05; Fig. 2A). Osteoblast cultured in a high glucose microenvironment showed the greatest apoptosis compared with 5.5 mM glucose group. Addition of glimepiride (10 µM) to the osteoblasts significantly reduced apoptosis compared with cells without glimepiride in both 5.5 mM and 16.5 mM glucouse groups. (Fig. 2B). Cyclin D1 involved in major cellular programs such as proliferation, migration and apoptosis, often give rise to splice isoforms with distinct biological activities. Cyclin D1 drives the G1-to-S phase progression of the cell cycle [21]. In the present study, glimepiride treatment resulted in significantly increased mRNA expression of cyclin D1 in both the control group and the 16.5 mM glucose group (Fig. 2C). Thus, we further investigated whether glimepiride affected the osteogenic differentiation potential of osteoblasts in the high glucose microenvironment. Alizarin Red S staining showed that the high levels of glucose significantly decreased Ca^2+^ accumulation in osteoblast cultures treated with 16.5 mM glucose, relative to the control group (Fig. 2D). Moreover, when 10 µM glimepiride was added to the 5.5 or 16.5 mM glucose culture medium for 72 h and the cells were then cultured for another 14 days, Ca^2+^ accumulation in both the control group and the 16.5 mM glucose group was markedly increased (*p*<0.05; Fig. 2D,E). Taken together, our results suggest that glimepiride induces proliferation and differentiation of rat osteoblasts in a 16.5 mM glucose microenvironment.

### Glimepiride affected P-eNOS expression by osteoblasts in a high glucose microenvironment

**Figure 3:**
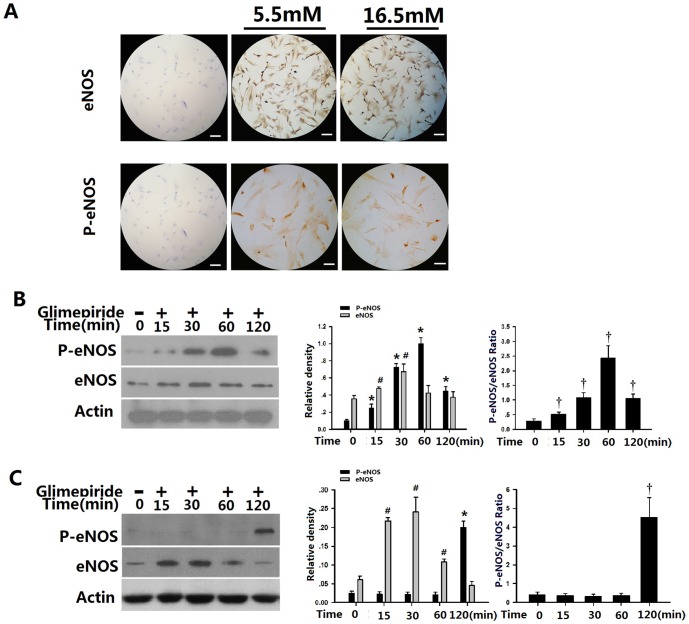
Effects of glimepiride on P-eNOS expression in rat osteoblasts. **A**, Immunohistochemical staining showed that osteoblasts expressed P-eNOS. Scale bar represents 100 µm. Osteoblasts grown with 5.5 mM glucose (control group; **B**) or 16.5 mM glucose (**C**) were incubated with glimepiride (10 µmol/L) for up to 120 min. Western blotting was performed to detect P-eNOS and total eNOS. Actin served as the internal control. eNOS, p-eNOS and p-eNOS/eNOS ratio are the mean ±SEM of three separate experiments. ^*^ *p*<0.05 vs control group related to P-eNOS expression. ^#^ *p*<0.05 vs control group related to eNOS expression. ^†^ *p*<0.05 vs control group related to p-eNOS/eNOS ratio.

To investigate the mechanism of the effect of glimepiride on osteogenesis and proliferation, immunohistochemical staining and western blotting were first used to determine the eNOS and P- eNOS protein levels of osteoblasts in the high glucose microenvironment. The eNOS isoform seems to play a key role in regulating osteoblast activity and bone formation, because eNOS knockout mice have osteoporosis due to defective bone formation [22]. Our data demonstrate that activated eNOS and P- eNOS are expressed in the cytoplasm of rat osteoblasts cultured with the two different concentrations of glucose (Fig. 3A). To confirm whether glimepiride activates eNOS expression in rat osteoblasts, osteoblasts in basic medium (5.5 mM glucose) and in high glucose medium (16.5 mM glucose) were cultured with glimepiride for 15 min, 30 min, 60 min and 120 min. Western blot analysis was performed to determine P-eNOS and eNOS expression. Interestingly, we found that under 5.5 mM glucose conditions, expression of P-eNOS was rapidly induced by glimepiride, with a significant increase observed after 15 min; P-eNOS was further substantially increased after 30 min and 60 min. However, the expression of P-eNOS exhibit a reduced tendency after 60 min (Fig. 3B). Consistent with the result of P-eNOS, the p-eNOS/eNOS ratio was increased gradually, the highest ratio was 2.46 after 60 minutes of incubation (Fig. 3B). P-eNOS expression in osteoblasts cultured with the high level of glucose differed from that in the control group. Addition of glimepiride to the culture medium containing 16.5 mM glucose had markedly increased the expression of P-eNOS after 120 min (p<0.05; Fig. 3C). However, P-eNOS protein expression and the p-eNOS/eNOS ratio had no statistically significant after 15 min, 30 min and 60 min compared with the control group (p<0.05; Fig. 3C).

### Glimepiride affected the biological characteristics of osteoblasts in a high glucose microenvironment

**Figure 4:**
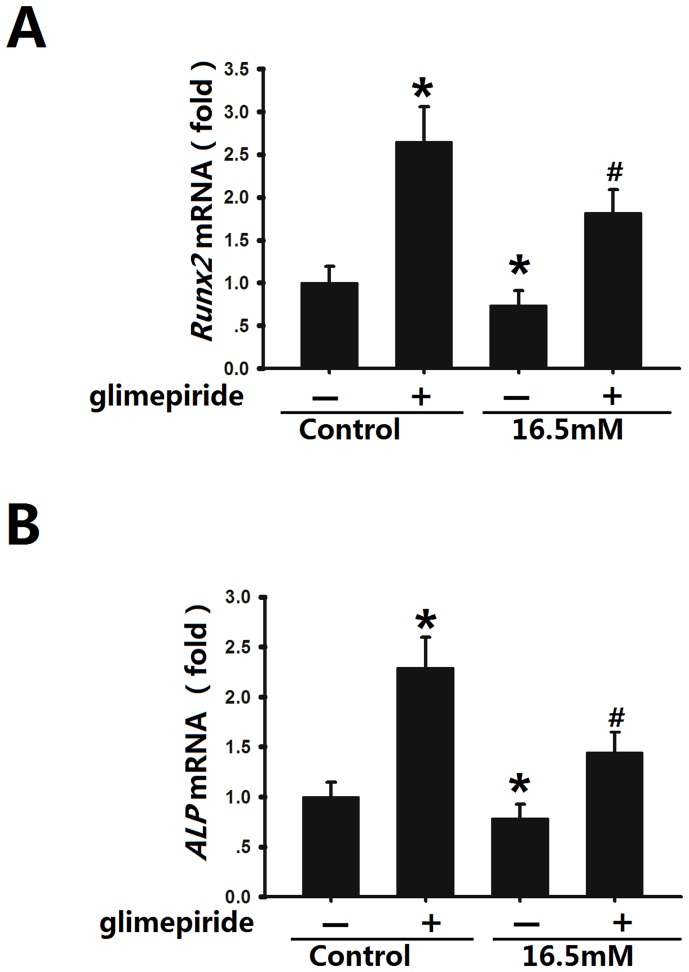
Effects of glimepiride on Runx2 and ALP mRNA levels in rat osteoblasts. Real-time PCR analysis of RUNX2 (**A**) and ALP (**B**) mRNA by osteoblasts stimulated with glimepiride for 120 min and then cultured with 5.5 mM or 16.6 mM glucose for 7 days. Expression levels were normalized to that of GAPDH. The results are the means (SD) of three independent experiments performed in triplicate. ^*^p<0.05 vs controls without glimepiride; # *p*<0.05 vs 16.5 mM glucose without glimepiride.

Osteoblasts grown in both 5.5 mM and high level glucose were treated with 10 µM glimepiride for 120 min. Furthermore, we extracted mRNA from both groups of osteoblasts cultured with basic media containing 5.5 mM or 16.5 mM glucose for 7 days and performed real-time PCR to determine the expression levels of osteoblast marker genes, including Runx2 (Runt-related transcription factor 2) (Fig. 4A), a critical transcription factor for osteoblastic differentiation [23], and ALP (Fig. 4B), an early osteoblast marker [24]. The expression of these marker genes increased by 2.62- and 2.25- fold, respectively, in the glimepiride treated group compared with the group without glimepiride (5.5 mM glucose, control group). The same trend of Runx2 and ALP mRNA expression was observed in osteoblasts cultured in the high glucose microenvironment (2.48- and 1.81- fold, respectively) (Fig. 4A,B).

### Suppression of the PI3K/Akt pathway by LY294002 suppressed expression of eNOS in osteoblasts

**Figure 5:**
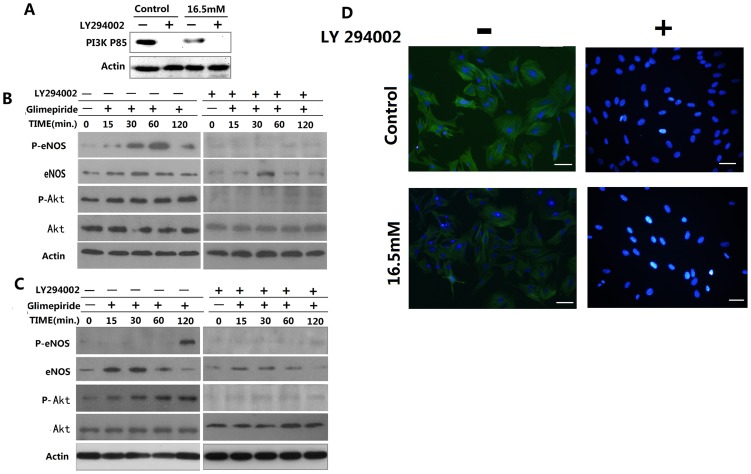
Effect of the PI3K inhibitor LY294002 on expression of eNOS in osteoblasts. **A**, Expression of PI3K P85 was measured by Western blotting in rat osteoblasts cultured with 5.5 mM or 16.5 mM glucose for 24 h and then changed to basic medium containing 10 µM LY294002 for 120 min. β-Actin was used as the control for equal loading. **B**, LY294002 inhibited glimepiride-induced phosphorylation of Akt and eNOS in osteoblasts cultured with 5.5 mM glucose. **C**, LY294002 inhibited glimepiride-induced phosphorylation of Akt and eNOS in osteoblasts cultured with 16.5 mM glucose. **D**, Immunostaining of P-eNOS in rat osteoblasts cultured with 5.5 mM or 16.5 mM glucose for 24 h and then changed to basic medium containing 10 µM LY294002 for 120 min.

To explore the effect of glimepiride on eNOS expression and the associated PI3K/Akt pathway in rat osteoblasts, we cultured cells in basic medium (5.5 mM) or basic medium containing 16.5 mM glucose, which efficiently inhibited osteogenic differentiation. LY294002 is a specific, reversible inhibitor of the adenosine triphosphate binding site of PI3K and has been widely used in investigating the functional and regulatory mechanisms mediated by PI3K. The two groups (control and 16.5 mM glucose) of osteoblasts exposed to 10 µM LY294002 resulted in inhibition of PI3K P85 as shown in Fig. 5A. The control group (5.5 mM glucose) was exposed to LY294002 for 0 min, 15 min, 30 min, 60 min and 120 min, after which cells were collected to determine the expression levels of P-eNOS and P-Akt. P-eNOS and P-Akt expression increased gradually from 15 min to 120 min in osteoblasts treated with glimepiride, but LY294002 abolished glimepiride-dependent P-Akt and P-eNOS levels (Fig. 5B). Osteoblasts cultured with 16.5 mM glucose, treated with glimepiride and harvested after 120 min exhibited increased P-eNOS and P-Akt levels (Fig. 5C). P-eNOS and P-Akt were inhibited in the glimepiride plus LY294002 group (Fig. 5C). To elucidate the mechanism of action of LY294002 on osteoblasts in the presence or absence of 5.5 mM glucose and 16.5 mM glucose, immunofluorescence staining was used to determine P-eNOS protein expression. e-NOS is downstream of the PI3K/Akt pathway and our results indicate that P-eNOS expression was inhibited in the cytoplasm of both groups of osteoblasts in the presence of LY294002 (Fig. 5D). These findings support the hypothesis that inhibition upstream of the PI3K/Akt pathway can inhibit the P-eNOS expression, which in turn suppresses osteoblast differentiation in high glucose microenvironments.

### Suppression of eNOS inhibited the osteogenic differentiation of osteoblasts in a high glucose microenvironment

**Figure 6:**
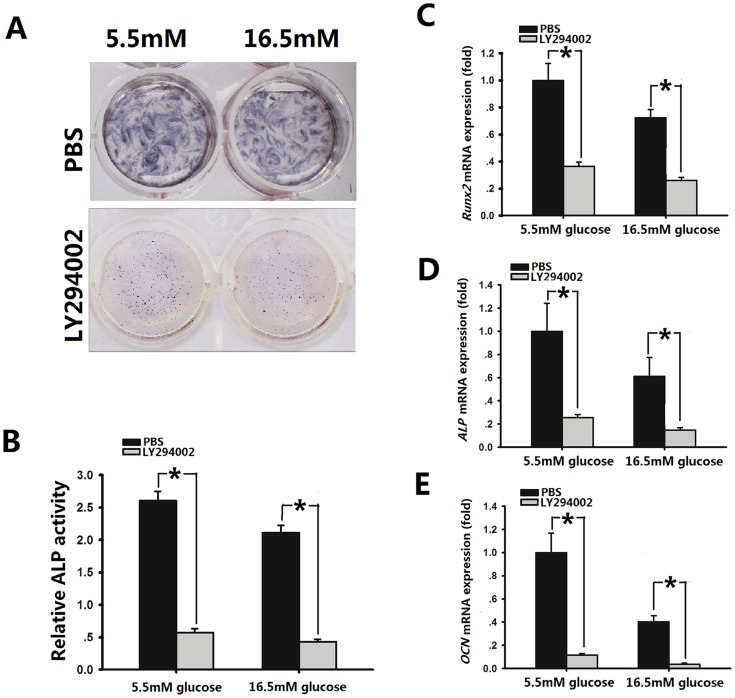
Effect of suppression of eNOS inhibited the osteogenic differentiation of osteoblasts. **A**, Scanned images of ALP staining of osteoblasts cultured with 5.5 mM or 16.5 mM glucose for 4 days and then changed to basic medium containing 10 µM LY294002 for 3 days. **B**, Quantitative evaluation of ALP activity. Data are the mean ±SD. ^*^ *p*<0.05 vs 5.5/16.5 mM+PBS group. Real-time PCR analysis of Runx2(**C**), ALP(**D**) and OCN(**E**) expression by osteoblasts cultured with 5.5 mM or 16.5 mM glucose for 24 h and then changed to basic medium containing 10 µM LY294002 for 7 days. Expression levels were normalized to that of GAPDH. Data are the means (SD) of three independent experiments performed in triplicate. ^*^ *p*<0.05 vs 5.5/16.5 mM+PBS group.

We examined the effect of LY294002 on the differentiation of osteoblasts. ALP staining was performed on cells cultured with 5.5 mM or 16.5 mM glucose treated with PBS(as the control group) and cells treated with 10 µM LY294002 (Fig. 6A). Osteogenic differentiation, which was assessed on the basis of ALP activity, was partially inhibited by 10 µM LY294002 in both 5.5 mM and 16.5 glucose groups (Fig. 6B). We then determined RUNX2, ALP and OCN mRNA expression in osteoblasts (5.5 mM and 16.5 mM glucose groups) treated with or without LY294002. In the 5.5 mM and 16.5 mM glucose groups the same trend was performed, expression of these genes was significantly reduced by LY294002 treatment compared with those without LY294002 (Fig. 6C,D,E), which was consistent with inhibition of the PI3K/Akt pathway acting to down-regulate these osteoblastic genes.

## Discussion

DM is associated with specific alterations of bone metabolism. Osteoblasts show promising potential for bone regeneration; therefore, it is of great interest to investigate changes in their proliferative ability and differentiation potential when cultured in differing microenvironments. We stimulated rat osteoblasts with 16.5 mM glucose and found that the hyperglycemic conditions interfered with the proliferation and mineralization of osteoblasts. Glimepiride is a third-generation sulfonylurea. In addition to its stimulatory effects on pancreatic insulin secretion, glimepiride has also been shown to play extrapancreatic roles. Our results demonstrated stimulatory effects of glimepiride on the proliferation and differentiation of rat osteoblasts, thus providing a possible mechanism for the beneficial effect of glimepiride on bone fracture, as reported by Vestergaard et al [25].

Our previous work suggests that glimepiride activates the PI3K/Akt pathway in osteoblasts. In endothelial cells, it is suggested that glimepiride induces eNOS phosphorylation with a dependent mechanism PI3k/Akt [8], [29]. Recent data from several groups show that eNOS is widely expressed on a constitutive basis in bone marrow stromal cells, osteoblasts and osteoclasts [26], [27]. Furthermore, some studies have revealed interplay between the PI3K/Akt pathway and the NO pathway, the latter of which is important for the proliferation and differentiation of osteoblasts. For instance, Afzal et al. suggest that impaired osteoblast proliferation and differentiation in the eNOS gene knockout mouse can be reversed by treatment with the NO donor potassium nitrosylpentachlorouthenate, indicating that disrupted osteoblast function is related to loss of NO-dependent signaling [28]. The results of the present study show that glimepiride activated Akt and eNOS phosphorylation in rat osteoblasts cultured with two different concentrations of glucose. In our study, Glimepiride increased the levels of both P-eNOS and total eNOS. Interestingly, Salani et al. [29] observed activation of P-eNOS, but not total eNOS, in glimepiride-treated endothelial cells, perhaps reflecting cell type specificity.

PI3K is a heterodimeric enzyme important for proliferation and apoptosis, while Akt is a downstream serine-threonine kinase that transmits survival signals from growth factors [30]. Akt activates eNOS, which leads to NO production [31]. Evidence shows that high glucose-induced apoptosis in human umbilical vein endothelial cells is mediated by sequential activation of c-Jun N-terminal kinase and caspase, and prevented by exogenous NO [32]. Activation of the ROS/PI3K/Akt/eNOS signaling pathway in early phase exerts protective effects against the induction of apoptosis by high glucose concentrations [33]. In our present study, we found that osteoblasts cultured in a high glucose microenvironment exhibited less proliferation and greater apoptosis than the control group. However, glimepiride stimulated cell proliferation and inhibited apoptosis in a high glucose microenvironment. Our results are consistent with our previous findings, considering that PI3K/Akt pathway is likely to be required for glimepiride to stimulate the proliferation of rat osteoblasts [16]. We also examined the mRNA expression of Cyclin D1, which is known to play a major role in cell proliferation and apoptosis in many cell types [21]. Cyclin D1 drives the G1-to-S phase progression of the cell cycle. We also examined the effect of glimepiride on Cyclin D1 mRNA level. We found that glimepiride treatment resulted in significantly increased mRNA expression of cyclin D1 in both the control group and the 16.6 mM glucose group. We hypothesized that the effect of glimepiride on rats osteoblast proliferation and apoptosis might be associated with cyclin D1, and need to be identified in future studies.

ALP is an early osteoblast marker and Runx2 is a critical transcription factor for osteoblastic differentiation [22], [23], We found that hyperglycemia interfered with the differentiation of rat osteoblasts, and glimepiride increased the expression of these marker genes at two different glucose concentrations. However, we showed that inhibition of PI3K P85 by LY294002 reduced ALP, Runx2 and OCN mRNA expression in osteoblasts. We conducted experiments using LY294002 at the concentration of 10 µmol/L as described in previous studies [4]–[6], [18]–[20]. Thomas et al [39] have shown that, at this concentration, LY294002 was nontoxic for osteoblastic cells, this conclusion has also been confirmed in our previous study [15]. Our results also indicate that P-eNOS expression was inhibited in the cytoplasm of both groups of osteoblasts in the presence of LY294002. These findings provide an additional mechanism for NO-elicited protection on the differentiation of rat osteoblasts. Our results concerning the complicated molecular relationship between eNOS and the PI3K/Akt pathway might explain the functions of glimepiride in osteoblasts under high glucose conditions. Some investigators have shown that NO donors and NOS inhibitors have little effect on osteoblast growth or differentiation, except at high concentrations where inhibitory effects were observed [34].

However, we found that PI3K P85 protein expression was markedly reduced at 16.5 mM glucose compared with 5.5 mM glucose, and we also noticed that eNOS and P-eNOS were suppressed in the group treated with 16.5 mM glucose. This finding is consistent with a previous report that showed that apoptosis in high glucose-induced umbilical vein endothelial cells occurs via reduced phosphorylation of eNOS with subsequent NO production [33], [35], [36]. Our previous studies also found that hat p-Akt expression was markedly reduced at 16.5 mM glucose compared with 5.5 mM glucose. We supposed that high glucose concentration suppressed PI3K/Akt/eNOS pathway. We also noticed that the increase in p-eNOS stimulated by the addition of glimepiride was lower at 16.5 mM glucose than that at 5.5 mM glucose. Interestingly, expression of P-eNOS induced by glimepiride was more rapidly under 5.5 mM glucose conditions than that 16.5 mM glucose conditions (after 15 min vs. after 120 min). Our previous studies also noticed that the increase in p-Akt stimulated by the addition of glimepiride was 27.32% lower at 16.5 mM glucose than that at 5.5 mM glucose. Kurowski et al and Oku et al reported that a high glucose concentration reduced p-Akt levels and reduced the insulin-induced Akt activation in skeletal muscles [37], [38]. We considered that hyperglycemia impaired glimepiride-stimulated phosphorylation and activation of Akt and eNOS. We also noticed that the increase in Runx2 and ALP mRNA stimulated by the addition of glimepiride was 16.4% and 37.02% higher at 5.5 mM glucose than that at 16.5 mM glucose. We supposed that the difference of rat osteoblastic differentiation between high and normal glucose conditions is related to glimepiride-mediated PI3K/Akt/eNOS pathway. However, further studies are needed to confirm these findings.

In our study, we found that hyperglycemia interfered with the proliferation, differentiation in rat osteoblasts. For the first time, we reported that glimepiride activated eNOS expression in rat osteoblasts cultured with two different concentrations of glucose, which the PI3K/Akt pathway plays a key role in this process. Our results also demonstrate that PI3K/Akt/eNOS pathway is likely to be required for glimepiride-induced differentiation of rat osteoblasts. However, the upstream signaling mechanisms involved in the up-regulation of eNOS expression by high glucose concentrations, and the contribution of endogenous NO production to preventing high glucose-induced cell apoptosis, need further research.
